# Impact of using authentic online learning environments on students’ perceived employability

**DOI:** 10.1007/s11423-022-10171-3

**Published:** 2022-12-02

**Authors:** María-Jesús Martínez-Argüelles, Dolors Plana-Erta, Àngels Fitó-Bertran

**Affiliations:** Universitat Oberta de Catalunya (UOC), Barcelona, Spain

**Keywords:** Employability, e-Learning, Higher education, Authentic learning scenario, Soft skills

## Abstract

The digitalization and globalization of society and the corresponding impact on the rules of the labor market is shifting the education sector toward new pedagogical approaches that integrate wholly online methodologies. Sustainable Development Goal 4 advocates for inclusive and equitable quality education that promotes lifelong learning opportunities, and, as we have seen during the COVID-19 lockdown, online learning can play a key role. In a context where lifelong learning becomes crucial to maintaining graduates’ employability, the innovative teaching methodologies that promote employable competencies in online environments are especially desirable. With the purpose of improving the employability of students, this article analyses the impact of introducing the Authentic Learning Scenarios (ALS) paradigm in an online environment. We develop a quasi-experimental design. Based on the nine ALS criteria and their application to e-learning, we redesign a course in a business degree program. Data from 135 students were collected, with special focus on achieving general competences. We compare the perception of the competency profile attained between a group of students who took the course before incorporating the ALS paradigm and another group that took it once it had been redesigned. Results show that redesigning the course enables students to perceive the learning process as more authentic, as well as acquiring a more advanced competence profile. Besides this, it has been shown that technology can contribute to building cognitive authenticity in virtual classrooms, without the need for face-to-face internships, which are often not a feasible option for students of online programs.

## Introduction

A key objective of the European Higher Education Area (EHEA) reform process is to boost higher education’s capability to improve graduate employability (Fitó et al., 2014; Prokou, 2008). This ability to produce employable graduates became even more critical after the start of the economic recession in 2008; since then, youth unemployment–recently aggravated by the impact of the COVID-19 pandemic–has become one of Europe’s major challenges, with graduate employability still dominating the higher education agenda (Jackson, 2013, 2014, 2016).

In May 2020, the third month marked by COVID-19 containment measures in most Member States, the seasonally adjusted unemployment rate was 7.4% in the Euro area. In the same month, youth unemployment in particular stood at 16% in the Eurozone (2267 million), with Spain having one of the highest rates (32.9%) (European Commission, 2020). This has kept young people at the forefront of the Europe 2020 Strategy for growth and jobs, a strategy based, among other things, on the idea that improvements in education should contribute to employability and reducing poverty.

Based on the European Commission's definition of employability, focused on competencies (European Commission, 2014, p. 67), employability should be aimed at improving the skills and knowledge of graduates. Thus, higher education programs and plans should ensure that graduates’ competency profiles increase their chances of employment. They should also bear in mind that employers perceive general competencies among graduates as key to their potential employability (De Weert, 2011; Dumford & Miller, 2017; Pitan, 2017).

A further factor is that technological progress and the changing needs of the labor market mean special attention must be placed on future employability and the importance of adaptability and transition between jobs over the course of people’s careers (Tymon, 2013). After the 2008 economic and financial crisis, European authorities reinforced the economic objectives of European lifelong learning policies and increased their political influence over the implementation of domestic lifelong learning reforms (Valiente et al., 2020). Technology brings both challenges and opportunities for lifelong learning, and the means to acquire the necessary specialization to bring profiles in line with these changing needs (Andronie & Andronie, 2014). As a means for lifelong learning, online education has a key role to play, as it is the predominant solution wherever education needs to be made compatible with full-time work (Berry & Hughes, 2019). Online learning models can serve particular niches. They provide access to education to students with certain physical, mental, or socioeconomic conditions, enhancing their employability (Devlin & McKay, 2016; Gill & Singh, 2019; Stone et al., 2016). Still, the challenges related to the digital divide persist (Littenberg-Tobias & Reich, 2020). Online education’s validity is further reinforced by the fact that its use in in-company training, and even traditional education, has grown, with the blended learning formats proving to be ever more common (largely driven by students themselves, now more at ease with using technologies).

As expressed in a World Economic Forum (2020) “The COVID-19 [pandemic] has resulted in a severe lockdown and schools shut all across the world. Globally, over 1.2 billion children are out of the classroom.” As a result, education has changed dramatically, with the distinctive rise of online learning, whereby teaching is undertaken remotely and on digital platforms. The latest research suggests that online learning has been shown to increase retention of information, and take less time, meaning the changes the coronavirus has caused might be here to stay. All the above attests to the increasingly important role of online education as a means of teaching. Therefore, any analysis of the effectiveness of education as a way to improve employability should also be analyzed in relation to how these educational processes can be undertaken in a technology-mediated context.

A number of studies state that innovative teaching methodologies are a requirement in any educational context for student employability (based on competency acquisition, and general competencies in particular). Several authors (Herrington & Herrington, 2006; Herrington et al., 2010) note that the use of Authentic Learning Scenarios (ALS) helps develop such competencies by bringing work experience closer to formal learning in higher education institutions.

Following the specific requirements of Herrington and Oliver (2000), there are few experiences of incorporating these authentic scenarios into online higher education environments, especially compared to the large number of experiences conducted in face-to-face education. This experience contributes to expanding existing studies in online environments and, in addition, it does so in a discipline not addressed until now, Business and Administration.

The ultimate objective of this research is to improve the employability of university graduates. To this end, this study focuses on examining the role of cognitive authenticity in the acquisition of general competencies considered key in employability. In order to do this, we redesigned a course from a fully online Business and Administration (BA) degree program, based on the precepts of authentic learning theory.

While professors improve the course to improve its authenticity, it is important to determine whether the student actually perceive that authenticity. In this regard, the first question to be answered is: (1) Do students perceive that the learning process for the course is developed within the context of an authentic learning scenario?

Secondly, in order to determine whether the ultimate objective improving graduate employability is achieved, the competence profile of the students is analyzed. Thus, the question to be answered is: (2) After studying the subject, does the student perceive a significant improvement in their level of general competencies that are key to employability?

The rest of the article is structured as follows: in "Education for employability" section, we describe the context in terms of both graduate employability competencies and authentic learning theory; in "Methodology" section, we present the methodology; in "Results" section, we present the main results; and in "Discussion and conclusion" section, we present and discuss the conclusions of the study.

## Education for employability

To answer both questions raised, we needed to determine the general competencies that actually boost graduate employability, and the attributes that characterize authentic learning.

### General employability competencies

The concept of employability is complex and encompasses many definitions and approaches. The employability definitions used in this article focus on graduates’ transition into the labor market after completing their higher education. From this perspective, according to a European Commission report (2014, pp. 67–68), employability can be approached in two ways: from a job-centered definition, i.e., the needs of the labor market, thereby stressing the demand side; and from a competency-centered definition, which places the emphasis on employable graduates and the supply side. Neither mutually exclusive nor alternative options, the two approaches can be seen as complementary in tackling graduate employability. However, although in practice it is difficult to separate the two perspectives, higher education institutions promote employability differently, depending on which approach they place greater importance upon. In this study, we explore the supply-side perspective, stressing the development of the general employability competencies that students need to acquire in higher education.

These competencies are, according to Yorke’s definition (2006), a set of achievements—skills, understandings, and personal attributes—that make graduates more likely to gain employment and be successful in their chosen occupations, benefiting themselves, the workforce, the community and the economy. Yorke stresses the quality and sustainability of work, while also focusing on future employability, understood as the ability to adapt to changes in professions.

However, as noted by Cinque (2016), programs in most European universities are still based on teaching traditional scientific competencies (or specific competencies) instead of paying more attention to soft or complementary skills. Soft skills (also referred to as complementary, transferable, or general skills, among other terms) are skills not related to a particular profession or specific course, but that help graduates find work and move around in the labor market (European Commission, 2014). Indeed, there seems to be a degree of consensus on giving greater weight to personal attributes rather than technical skills when taking labor market needs into account (Tymon, 2013). Studies conducted specifically among business graduates show that university curricula need to help graduates realize that their attitude towards work is just as important as the work itself (Hodges & Burchell, 2003); therefore, personal competencies and attributes are relevant factors for employers considering taking on business graduates (McMurray et al., 2016).

Although numerous sets and taxonomies exist in relation to general competencies and their relative importance, a definitive list of the most relevant competencies for higher education graduates has yet to be devised (Cinque, 2016). To respond to the changing needs of the current labor market and its projected evolution in the twenty-first century, this study adopts the taxonomy of 15 current employability skills produced by Ornellas et al. (2019), as shown in the Table 1.

**Table 1:** Taxonomy of employability skills required for new graduates by Ornellas et al. (2019)

	Skill	Definition
Cognitive	Analytical thinking	Gathering, analyzing and articulating information from different sources for solving problems and decision making
	Creative thinking	Thinking outside the box in order to bring new ideas to solve problems
	Foreign language	Intercultural understanding and performing in a language different from the native language
Methodological	Learning to learn	Effectively managing one’s own learning process and needs
	Problem solving	Engaging in the actions or thoughts necessaries to find solutions to a difficult or complex question or situation and resolve conceptual problems
	Decision making	Thinking of several choices, relevant information and predicting the consequences
	Digital skills	Being digitally competent in 4 areas (Vuorikari et al. 2016): information and data literacy; communication and collaboration; digital content creation; and safety
	Results-oriented performance	Ability to make organizational efforts according to the goals pursued (Haselberger et al., 2012)
	Self-management	Setting goals and priorities through the selection and distribution of tasks and resources. Time management, organization, responsibility, and self-reliance
Social	Communication and interpersonal skills	Articulating, transmitting, and effectively defending arguments, ideas, feelings, or information. Listening, understanding and being receptive to others
	Teamwork	Working collaboratively with others both face-to-face and online
	Cross-cultural and diversity competence	Working with people of different ethnicities, religions, cultural background, genders, etc
	Ability to cope with changes	Dealing with changes and uncertainty and adapting to new situations
	Conflict management	Taking control of a conflict between two or more parties in an assertive way Stress management
	Stress management	Showing endurance in complicated or stressful situations, workloads while maintaining the same quality level in the tasks accomplished (Haselberger et al., 2012)
	Course specific	The set of knowledge and abilities required to successfully perform a specific occupation (i.e., lawyer, accountant, and teacher)

Ornellas et al. (2019) put together this list of skills on the basis of a literature review analysis of recent international reports, frameworks, and studies, including Davies et al. (2011), Haselberger et al. (2012), and Humburg et al. (2013), among others. They went on to validate the final taxonomy by means of a questionnaire and discussion forum with representatives from the world of work, teachers, and university students from six institutions in three different European Union countries. As well as containing specific skills, this taxonomy places special emphasis on general social, methodological, and cognitive competencies. Ornellas et al. (2019) also highlight the importance of confirming the relevance of these competencies through additional empirical studies.

### Authentic scenario-based learning

Institutions of higher education around the world emphasize that their graduates should do more than simply reproduce discipline-specific knowledge and skills that form the core of most university courses (Barrie, 2007). There is a significant disconnect between the job-readiness of graduates and the expectations of their employers (Jackson, 2009). Companies often complain that graduates have plenty of knowledge but lack competence in the workplace (Bastiaens & Martens, 2000). Designers of learning activities for concept acquisition frequently ignore the influence of culture and context, failing to take into account the very situations in which students will learn or use these concepts, thus limiting the activities’ effectiveness (Brown et al., 1989; Heaviside et al., 2018; Kornelakis & Petrakaki, 2020).

The key to improving graduate employability lies in reducing the gap between the theoretical learning that takes place in formal education and the workplace in which graduates will apply this knowledge (Rule, 2006). As an alternative to conventional activities, Brown et al. (1989) proposed a model of cognitive learning, based on the inculturation of students in authentic practices through activity and social interaction. From this perspective, authenticity relies on neither the learner, the task nor the learning environment, but on dynamic interactions among all three (Barab et al., 2000). In this way, conceptual knowledge is connected to the situations where it is found.

Several authors have discussed how this type of learning should be developed. Huang (2002), for instance, stated that adult students learn better when presented with a real-life problem in a real-life context. Consequently, including internships in university programs is a good way of bringing university and professional knowledge together, while also improving employability.

However, other authors, such as Alessi (2000) or Herrington and Herrington (2006), show that maximum fidelity to the real-life context does not necessarily lead to maximum effectiveness in learning. According to this view, the cornerstone for learning design is cognitive authenticity (Herrington et al., 2003; Smith, 1987) rather than physical authenticity. In their model of situated cognition, Brown et al. (1989) argued that meaningful learning would only take place if it is embedded in the social and physical context within which it is used. In this regard, the proposed learning activities must reflect how students will apply the knowledge in practice when they are required to engage in authentic activities.

Moving from theory to practice in higher education is a great challenge, although the exploration of learning models in the literature may lead to a tacit understanding of the general characteristics, the actual implementation of these characteristics is a different matter altogether (Herrington et al., 2015). Many research studies have explored the integration of authentic learning into teaching and learning (Chiu et al., 2018; Herrington & Parker, 2013; Herrington et al., 2014; Matthew & Butler, 2017; Pu et al., 2016; Tabuenca et al., 2016). However, there is a need for guidelines that can help bring the quality of authenticity in learning into the classroom, enable assessment of the extent to which this objective is achieved, and suggest means of improvement. In this respect, Herrington and Oliver (2000), based on a literature review, identify nine critical characteristics of an authentic learning environment:

1. Provide an authentic context that reflects the way the knowledge will be used in real-life.
2. Provide authentic activities and tasks.
3. Provide access to expert performances and the modelling of processes.
4. Provide multiple roles and perspectives.
5. Support the collaborative construction of knowledge.
6. Promote reflection.
7. Promote articulation.
8. Provide coaching and scaffolding.
9. Provide for authentic assessment of learning within the tasks.

According to these authors, environments that include these elements are more suited to facilitating authentic learning. The same article provides instructions for operationalizing the elements identified when designing a learning environment.

Among the many articles in the literature that cite the work of Herrington and Oliver (2000), we have focused on those that explicitly demonstrate the practical implementation of elements of authentic learning in higher education in a non-face-to-face environment. We consider that an online environment in which students and teachers do not coincide in time and/or space, and in which a substantial part of the teaching and learning activities—80% or more, according to Allen (2007)—is created with information technologies and internet-based communication. The Table 2 summarizes the relevant aspects of the studies selected.

**Table 2:** Articles that implement the principles of Herrington and Oliver (2000) in higher education and in a non-face-to-face environment

Authors	Objective	Education/institution/ program-department/course	Analysis	Conclusions
Leppisaari et al. (2013)	Examine the implementation of authenticity using Herrington et al.’s (2000) authentic learning elements	Education: Online Institutions: Eight higher education institutions from five countries (Finland, South Korea, Belgium, Wales, and Canada) Course: Applied Sciences courses	Qualitative with self-evaluation and comparative evaluation by pairs of 12 teachers and an authentic learning expert	- Successful implementation. Relevant cultural factors in authentic online learning - Best-implemented elements: multiple roles and perspectives, and authentic coaching. Weak implementation: collaborative construction of knowledge and reflection
Rowe et al. (2013)	Examine whether authentic learning elements, and Google Drive for interaction, enable the development of critical thinking among students	Education: Blended Institution: University of Western Cape in South Africa Department/course: Physiotherapy Department / Applied Physiotherapy	Qualitative, based on two focus groups. Twelve students took part	- Student transformation in terms of how they perceive critical attitudes to knowledge and authority - Authentic activities and technological platforms are a good tandem for developing critical thinking
Rowe (2016)	Determine whether authentic learning principles enable the development of generic attributes that prepare students as active agents of social wellbeing	Education: Online Institution: University of Western Cape in South Africa Department/course: Physiotherapy Department / Professional Ethics	Qualitative. Two focus groups of four students each, and two group interviews with two students each	- Development of a subset of generic attributes: internal motivation; challenging knowledge and authority; and empathetic communication - Framework of well-applied authentic learning facilitates the development of generic attributes in the graduates
Luo et al. (2017)	Evaluate the use of Herrington et al.’s (2003) framework in the design of a course to train pre-service teachers in the use of technological applications	Education: Blended Institution: Midwestern university in the United States Program: Pre-service teachers seeking licensure to teach in K-12	Design-based research Preand post-survey, and a personal blog, with 48 students	- Good student perception of the course design - The activities were so realistic that they could be used once the participants became teachers
Teras and Kartoglu (2018)	Discover whether the design and implementation of authentic learning translates into a professional learning experience for the student	Education: Online Institution: World Health Organization Course: e-VVM, vaccine management course	Grounded theory approach to the research task. Semi-structured interviews with seven students, and observations on the online learning environment	- Raised perception of authenticity - Authentic learning elements useful for design of Online Professional Development - Authentic learning promotes other abilities: work as well as possible, invest time and effort in tasks, and maintain collaboration subsequent to completion of the course

Empirical studies show that Herrington and Oliver’s (2000) nine elements provide educators working in online environments with a useful guideline for creating an authentic learning experience in different courses and disciplines. In authentic learning, the emphasis lies with the design of authentic tasks that deliberately maintain the complexity of real-world problems (Teras & Kartoglu, 2018). Therefore, it is necessary to take the context into consideration before implementing and analyzing a design that could be successful.

A common aspect in the five studies is the essential role of technology in the re-creation of more authentic environments. As Teras and Kartoglu (2018) argued, authenticity is promoted by using technology as a cognitive tool for problem-solving, collaborating and constructing knowledge. We could say that technological tools function as a support to learning and are not an end in themselves.

In the study of Leppisaari et al. (2013), authenticity is analyzed from the teachers’ perspective without contrasting the effectiveness of the design with the students. Even if the will of teachers and educational designers is authenticity, it is necessary for students to share the perception of authenticity for authentic learning to reach its potential, as Gulikers et al. (2008) recommended. It is interesting to note that, for the teacher, the implementation of nine elements constitutes a challenge because it requires the learning to be designed with a more holistic vision that goes beyond merely teaching the knowledge required by the discipline to enable the inculturation of the student in a relevant context. Although teachers may be prepared in terms of knowledge, attitude, and interest to use an authentic learning approach, they may lack the skills to fully implement it (Baskaran & Abdullah, 2020). In this respect, Leppisaari et al. (2013), for example, relied on an expert to guide them through the process of implementation of authentic learning elements.

Based on the success of previous studies, we consider incorporating authentic learning scenarios into the design of online teaching to be a highly significant step. In particular, given the importance of online learning for lifelong employment. One of the areas in which the presence of job offers is more frequent is Business Management and Administration. Therefore, we consider it appropriate to analyze whether it was possible to increase the authenticity of the courses in this discipline. A discipline in which the impact of online ALS has not been previously analyzed. We conduct this evaluation from the students’ perspective, as suggested by Gulikers et al. (2008).

Based on the nine elements identified by Herrington and Oliver (2000), we focused on the redesign of a course of the Business Administration and Management degree from a totally virtual university. Considering that our objective is to improve the level of employability of university graduates, this research focuses on determining the role of ALS in acquiring the general competencies considered key for students’ employability.

## Methodology

A quasi-experiment was conducted within an online environment to answer the two research questions: (1) Do students perceive that the learning process for the course is developed within the context of an authentic learning scenario? and (2) After studying the subject, does the student perceive a significant improvement in their level of general competencies that are key to employability?

**Fig. 1:**
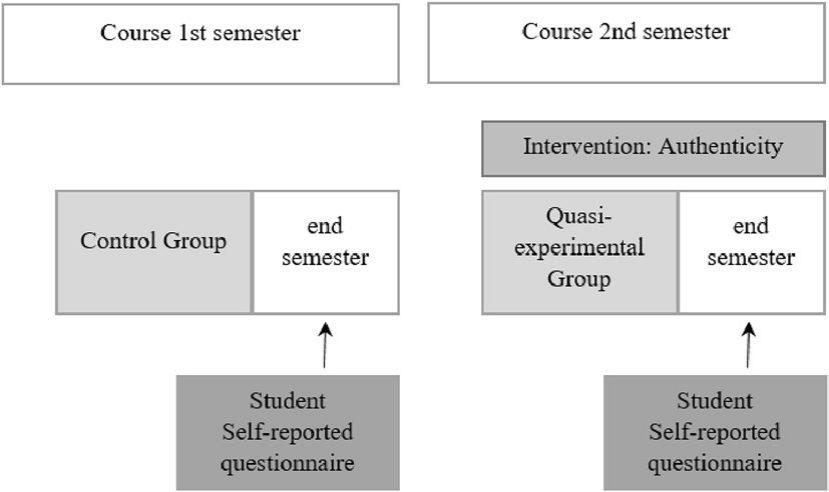
Quasi-experiment procedure

The experimental situation (see Fig. 1) consisted of redesigning the dynamics and activities of the course to increase its cognitive authenticity. This design was applied to all students enrolled in the subject in the second semester of the course (quasi-experimental group) and the students enrolled in the previous semester were used as the control group.

Some of the experimental conditions were controlled: the technological environment used by the students in the two semesters was identical, as well as the pedagogical model, the professors were the same and the students in the sample had similar characteristics (age, gender, and work experience in the discipline).

Questionnaires were sent at the end of each semester, one in January (for students in the control group) and one in June (for students in the quasi-experimental group), enabling a comparison between the two groups of the general competencies acquired by the students.

### The online learning context

The study was conducted at the Universitat Oberta de Catalunya (UOC). It is a fully online and asynchronous university. Due to its history and size, the UOC can be considered a representative case of a major online university.

The experiment was applied to a course on the bachelor’s degree in Business and Administration (BA). The BA degree, like all UOC programs, has a user-centered activity-based educational model. The whole learning process takes place through its virtual learning environment, comprising a learning management system (LMS), learning materials, a digital syllabus, and assessment tools. Assessment is continuous and formative and has an impact on students’ learning, helping them to continuously improve, with assessment activities helping to ensure that learning objectives are achieved, and skills developed. The characteristics of the pedagogical model, which has remained unchanged in the redesign of the course, respond to the formative needs of part-time students who mostly combine work with training. The work dynamics provide for the continued accompaniment of an expert teacher in the field that promotes and evaluates the achievement of learning outcomes. The particularities of this pedagogical model and the existence of an LMS that integrate learning activities, resources and tools, communication spaces, and evaluation into a virtual classroom favor the online application of authentic learning scenarios (ALS). The BA bachelor’s degree program has over 5,000 students, one of the University’s most popular programs. Similar programs are offered at many universities around the world and the degree is in high demand in the labor market. Specifically, the research was conducted with students who took the Financial Statement Analysis course, which is one of the program's compulsory advanced courses. The learning outcomes for students taking the course are particularly relevant to one of the most common professional profiles for BA graduates, that of assistant finance manager. Two specific competencies are needed to work in this position: (1) the ability to generate relevant economic knowledge from data, applying the appropriate technical instruments, and (2) the ability to efficiently manage a company, considering its competitive situation and identifying its strengths and weaknesses.

### Participants

As can be observed in Table 3, 354 students took the course in the first semester and 329 students in the second semester. A total of 86 students from the control group answered the questionnaire (46% women) with a sample error of 9.15% at a 95% confidence interval (CI). In the quasi-experimental group, 49 students answered (47% women) with a sample error of 10% at a 95% CI. Regarding age, the percentages were similar for both groups. In the control group, 41.9% were under 35, while 42.9% of the quasi-experimental group were in this age range. The age and gender descriptions were similar in both samples, and between 60 and 70% of the students who responded held jobs related to their studies.

**Table 3:** Characteristics of the control and quasi-experimental groups

Academic year 2017/18	Students	Sample	Gender		Age		Job related to studies
			W	M	< 35	≥ 35	
1st semester (control group)	354	86	46	54	41.9	58.1	69.8
2nd semester (quasi-experimental group)	329	49	47	53	42.9	57.1	59.2

### The “authenticity” of the course

In the following subsection, we will explain that we measure authenticity according to the nine authentic learning criteria identified by Herrington and Oliver (2000). We redesigned the course following the directives and examples for developing and implementing authentic learning tasks in online environments included in Herrington’s et al. guide (2010). Table 4 briefly explains how the nine authenticity criteria have been incorporated.

**Table 4:** Incorporating ALS elements by redesigning activities

*Provide an authentic context*
We recreated a realistic situation in which the student had just been hired as an assistant financial manager. The financial manager assigned responsibility for preparation of the company’s financial report to the new assistant manager; the report would be presented at the next meeting of the Board of Directors. They agreed on periodic meetings to review the progress of the report. The initial conversation and periodic meetings were recreated on video
*Provide authentic activities*
The student had to produce an authentic report by engaging in complex activities and performing an analysis of different real-life statements and indicators. The financial manager also formulated questions and offered guidance, generating interaction with the student
*Provide access to experts*
The course instructors are experts in the field and, acting as financial managers, gave continuous feedback and guidance to the students, who could interact at any time and have access to similar reports prepared by experts
*Provide multiple roles and perspectives*
In relation to each part of the report, the financial manager would raise difficult questions about the impact of the conclusions obtained on the different departments of the company (e.g., marketing, production, human resources)
*Support collaboration in the construction of knowledge*
Collaboration was particularly focused on the interaction with the “boss,” who assigned the responsibility for the report and gave continuous feedback. Students could also interact with other students
*Promote reflection*
Student reflection is key to transforming the information provided by the financial statements into a SWOT analysis of the company
*Promote articulation*
During the course, the student had to articulate a complete report on the company’s financial situation and conclude with a SWOT analysis, as well as respond to the “awkward” questions raised
*Provide coaching and scaffolding*
The course instructor, as the student’s “boss,” had a clearly defined role as the student’s coach, providing continuous feedback and guidance to the student
*Provide for authentic assessment of learning within the tasks*
Continuous assessment of the different stages of the report was provided, including grading and personalized feedback at each stage

The course’s classroom dynamic and the students’ learning activities focused on performing realistic, complex tasks. The first step was to identify in which job position the learning result of the course would be applicable (i.e. undertaking a financial diagnosis of the company’s financial statements). This diagnostic task is common within the finance departments of companies, so we decided that students would adopt the role of assistant finance managers. A very plausible job for a BA graduate. As well, and to increase the level of perceived realism, we decided that the students would be recent BA graduates that had just joined a company. Their superior, the course instructor, acting as the company’s financial manager, entrusted them with producing a professional financial report on the company’s economic and financial situation, which the financial manager would then present at the next board of directors meeting.

**Fig. 2:**
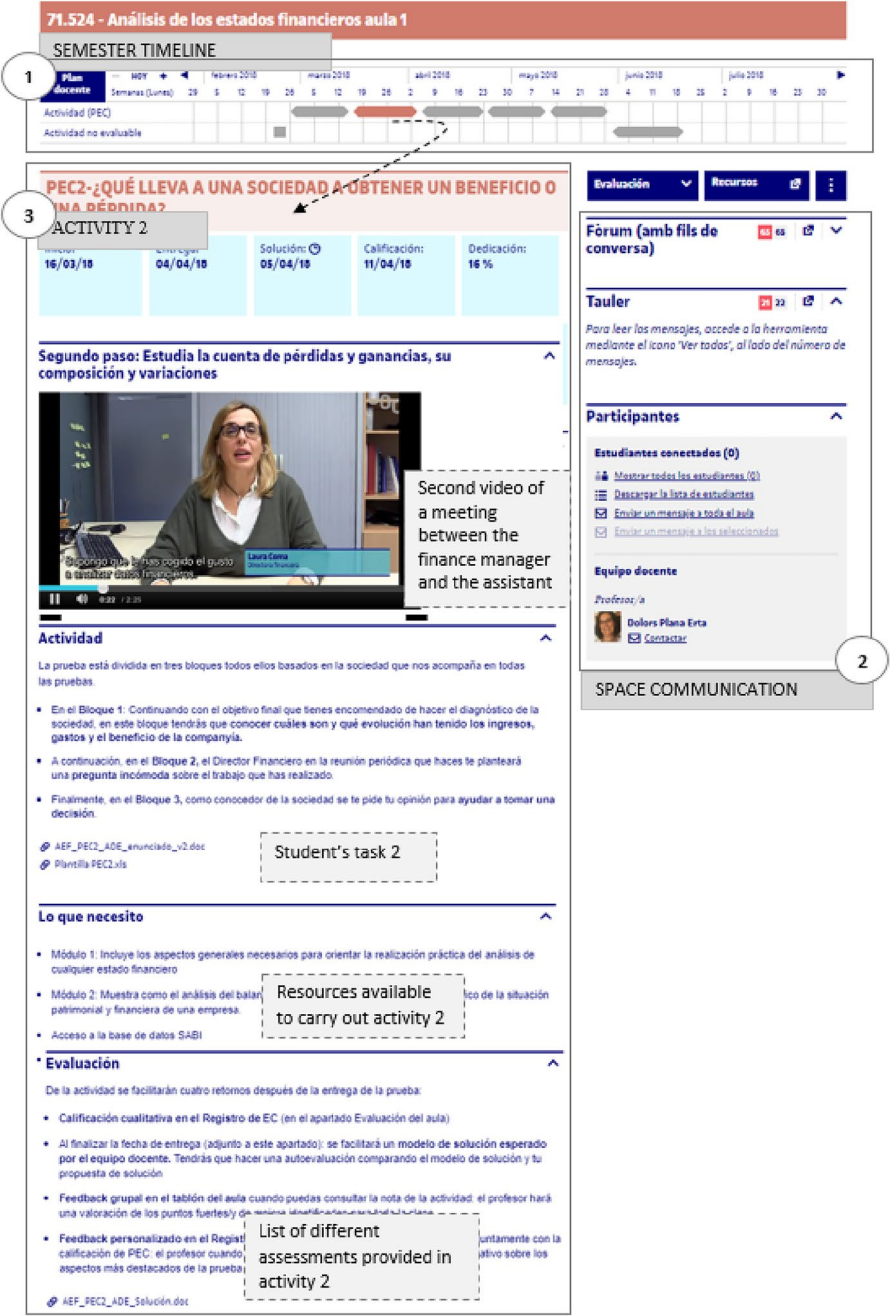
Parts of the online classroom. Second activity

Once defined the task to be conducted by the student, the roles, and the context where it would be conducted, the second step was to implement it in the virtual classroom. The virtual classroom integrates all the necessary elements so that the student can have an immersive experience. As shown in Fig. 2, the virtual classroom is divided into three main sections: (1) Upper part: timeline showing the start and delivery dates of the activities; (2) Right side: space for the teacher-student communication and for student–student communication, with the “board” and “forum” and other possible resources; and (3) The central part (changing for each activity): with information, resources and tools that are necessary for the correct development of the current activity.

In the implementation, the following aspects of the business reality were considered: (a) in a job there is a boss/superior who assigns the tasks; (b) a recently graduated employee must receive clear general instructions on the task to be performed, and have supervision to correct deviations from the initial objective; c) complex tasks are not immediate, such as the diagnosis of the economic-financial situation, and require a long time of dedication, and (d) whoever performs a task is responsible for the obtained result before their superior.

The learning environment was online and asynchronous. The entire relationship between student and teacher took place in the virtual classroom, without moments of synchronicity. Therefore, it was decided to recreate the meetings between the course instructor/company financial manager and the recently hired student/assistant finance manager through six videos embedded in the central part of the virtual classroom (see Fig. 2).

The videos lasted about 3 min and were provided to the student every 18–20 days. Table 5 shows the objective that was pursued in each video in relation to the design of the course.

**Table 5:** List of videos, course activities and the established objective

Videos	Activity	Meeting objective
Video 1	Course presentation	1st meeting: Contact. First day of work for the assistant, where they meet their superior, the financial manager. The manager explains what the task will be that they must complete in the coming months (report) and how they will be organized
Videos 2 to 5	1,2,3,4	4 control meetings. Every 18–20 days a meeting is held with the financial manager. The manager reviews the progress of the work and tells the assistant what the next activity to deliver would be
Video 6	5	Last meeting. Financial manager congratulates the assistant for the work done up to that moment. The boss calls on them to prepare the final report, for the board meeting, based on the four preceding activities

Students were required to conduct four learning activities individually; these activities would provide the theoretical and practical basis for completing the last activity, the final financial report. To conduct the sectorial comparison of the analyzed company, the student had to use a professional database (Iberian balance analysis system, SABI) as it would be done in a real business context. Access to this tool was available from the virtual classroom itself.

In order to expose students to a range of roles and perspectives, in each of the four activities the financial manager placed the assistant in an awkward position by asking their opinion about impending decisions affecting other company departments, such as general management, production, human resources, or marketing. For instance, in Activity 1, the financial director asks the assistant: “I met with the Sales Manager yesterday and he is very interested in entering a new market that might lead to a big rise in sales. But he’s a little afraid because he doesn’t know enough about the payment behavior of these new customers and was thinking about contracting non-recourse credit insurance. He’s shown me a proposal from an insurance company made to him a few months ago, whereby, for a volume of sales of 30,000,000 euros a year (forecasted rise in sales), they offer credit insurance to cover a maximum of 80% of the amount sold to the customer. I need you to analyze the proposal, consider the costs of the two alternatives (insuring or not insuring) and give me the reasons for your choice so I can convey them to the Sales Manager.”

Assessment was continuous over the semester. Students were given a mark for each activity, along with a personalized comment they could use to improve the final report.

### Measurement instruments

The measurement of the indicators of general employability competencies and authentic learning were reported by the students through questionnaires given online (student self-reported questionnaire). The design of the questionnaire was based on the bibliographic review reported in the education for employability section.

The questionnaire was divided into three parts. (1) Demographic data (age, gender, work experience, between others). (2) A set of seventeen questions related to the competencies acquired in the course. Questions to know to what extent the students perceived that the course helped them to acquire the specific competencies of the course (2 questions), as well as general key employability competencies (15 questions) identified by Ornellas et al. (2019). Students responded according to a 5-point Likert scale, ranging from 1 = “Not at all” to 5 = “A lot”.

Also, we asked students to select from the fifteen general competencies the five that they perceived as most important in relation to their professional field. We consider the students’ opinion particularly well-informed on this point because, as mentioned above, most of them were already working in jobs related to BA graduates.

(3) Finally, a set of 16 questions related to authentic learning was established. These questions were to assess compliance with the nine elements that characterize authentic learning according to Herrington and Oliver (2000). As can be seen in the first column in Table 6, one or more questions assessed the degree of authenticity in the learning environment for each of the nine elements. Questions were obtained from the questionnaire by Ornellas et al. (2019), that was based on the guidance for the implementation of ALS by Herrington et al., (2010, pp. 76–78). In this block, students responded according to a 5-point Likert scale, ranging from 1 = “Strongly disagree” to 5 = “Strongly agree”.

### Data analysis

Data analysis was conducted using two basic procedures: (a) univariate descriptive statistics and (b) bivariate inferential statistics. The statistical analysis was conducted with SPSS.

Regarding point (a), univariate descriptive statistics were used producing basic descriptors as means (M) and standard deviations (SD). In relation to point (b), through a bivariate inferential analysis, a means comparison was made between variables of the control group and the quasi-experimental group. Before the hypothesis contrast was addressed, the Kolmogorov–Smirnov (K-S) normality test was performed, rejecting the null hypothesis. Thus, in relation point (b), through a bivariate inferential analysis, a means comparison was made between variables of the control group and the quasi-experimental group. Before the hypothesis contrast was addressed, the Kolmogorov–Smirnov (K-S) normality test was performed, rejecting the null hypothesis. Thus, we ran non-parametric statistics on independent samples (Mann–Whitney U test).

## Results

### Do students perceive that the learning process for the course is developed within the context of an authentic learning scenario?

Given the results in Table 6, this question may be answered in the affirmative. Significant differences were found between the perceptions of mean levels of authenticity between the students in the control group and the group of students in the redesigned course. The redesign of the course considerably improved students’ perception of the degree of authenticity of the course’s learning environment (in the professional context of an assistant financial manager): students in the quasi-experimental group gave a rating of over 4 (on a 5-point scale) for 75% of the attributes, compared to only 25% in the control group.

**Table 6:** Authentic learning attributes statistics

E^a^	Basic concepts behind the questions to assess the nine authentic learning attributes	Quasi-experimental *M* (SD)	Control *M* (SD)	Mann–Whitney U test
1	Course presented a real-life context	4.49 (.51)	4.08 (.83)	.004*
2	Real-life tasks	4.39 (.61)	4.02 (.91)	.026*
2	Tasks involved complex problems	4.16 (.75)	3.76 (.93)	.017*
2	Adequate choice of information	4.39 (.70)	3.84 (.93)	.000*
2	Externalizable tasks and strategies	4.39 (.53)	3.90 (.78)	.000*
3	Access to expert knowledge	4.24 (.88)	3.36 (1.12)	.000*
4	Different viewpoints	4.12 (.73)	3.55 (1.05)	.001*
5	Group rather than individual marks	3.10 (1.39)	1,81 (1,08)	.000*
6	Decision-making to complete task	4.20 (.87)	3.24 (1.08)	.000*
6	Expert comparison	3.82 (1.11)	2.94 (1.31)	.000*
6	Reflection group	2.94 (1.38)	1.87 (1.06)	.000*
7	Tasks enabled debate	3.80 (1.04)	3.20 (1.24)	.004*
8	Teacher support	4.27 (.86)	4.10 (1.02)	.417
9	Performance polished	4.12 (.70)	4.19 (.86)	.365
9	Long-term activity periods	4.16 (.90)	3.85 (.98)	.037*
9	Multiple assessment	4.12 (.75)	3.27 (1.21)	.000

*M* mean, *SD* standard deviation

^a^Elements: The number in this column indicates which of the nine critical elements identified by Herrington and Oliver (2000) it refers to. These elements were assessed by a set of questions. The second column only includes the essence of the question, not the original in the survey

*p < *.01*

The differences were significant for all but two of the attributes. The non-significant results for these two specific attributes -teacher support and performance polished- were not thought to invalidate the overall results. The mean for teaching support rose from *M* = 4.10 to *M* = 4.27, although it did not increase at a significant level. While the redesign did not aim to increase the already elevated level of teaching support, students did perceive an improvement in said level. The polishing and refinement of the activities remains at high levels, although the mean dropped slightly (albeit not significantly, from *M* = 4.12 to *M* = 4.19). This attribute was one of the three that in the survey were related to the ninth element of Herrington and Oliver (2000), regarding the provision of authentic assessment of learning within the task. The other two attributes–long-term periods and multiple assessment–experienced a significant improvement when redesigning the course.

Therefore, we consider that the objective of improving the ninth element of authenticity was met, in global terms.

In short, the redesigned course was perceived as a course that provided a greater degree of authentic context, authentic activities, access to experts, multiple roles and perspectives, support in the construction of knowledge, promotion of reflection and articulation, and authentic assessment.

Therefore, the results showed that students perceived the learning context proposed by the course as realistic and a cognitive replica of a real-life work scenario. They also perceived the activities as real-life tasks similar to challenges that students were likely to face in the world of work.

### Does the student perceive a significant improvement in their level of general competencies that are key to employability?

Table 7 shows that students in the experimental group perceived an improvement in almost all the general employability competencies identified by Ornellas et al. (2019), with means of over 3 (on a scale 5) for 80% of the competencies, compared to only 53% over 3 in the control group. The improvements were statistically significant for all competencies, except results orientation. It should be borne in mind that the original course design was already highly results-orientated (*M* = 4.08), hence the improvement was not particularly significant (*M* = 4.22). In addition, the increase in general competencies was not detrimental to the two specific competencies of the course; in fact, their means also improved (*M* = 4.41 to *M* = 4.61 in one, and *M* = 4.19 to *M* = 4.55 in the other) for p-values under .05.

**Table 7:** Statistics for perceived improvement in general employability competencies, in order of importance, as attributed by students

Employability competency	Importance attributed competency % total	Quasi-experimental *M* (SD)	Control *M* (SD)	Mann–Whitney U test
Analytical thinking	14.1	4.55 (.94)	4.15 (.78)	.000*
Problem-solving	12.5	4.27 (.93)	3.77 (.86)	.000*
Decision-making	9.9	4.31 (1.14)	3.92 (.88)	.000*
Teamwork	9.7	2.69 (1.53)	1.80 (.92)	.010*
Foreign language	7.3	2.57 (1.56)	1.56 (.81)	.000*
Communication and interpersonal skills	6.4	3.53 (1.28)	2.67 (1.21)	.000*
Digital skills	5.9	3.90 (1.16)	2.92 (1.11)	.000*
Self-management	5.9	4.31 (.92)	3.99 (.91)	.018*
Results orientation	5.7	4.22 (.80)	4.08 (.76)	.216
Ability to cope with changes	5.1	3.67 (1.27)	3.02 (1.26)	.003*
Creative thinking	4.8	3.96 (.98)	3.26 (1.05)	.000*
Stress management	4.0	3.59 (1.27)	2.50 (1.31)	.000*
Conflict management	3.5	3.49 (1.37)	2.51 (1.25)	.000*
Learning to learn	3.1	4.12 (1.03)	3.40 (1.09)	.000*
Cross-cultural and diversity competency	1.1	2.98 (1.42)	2.51 (1.18)	.047*

*M* mean, *SD* standard deviation

*p < *.01*

Moreover, the students felt they had significantly improved in the five general competencies they considered most relevant to the employability of a BA graduate, as indicated in Table 7: analytical thinking, problem-solving, decision-making, teamwork, and foreign language. The last two of these, incidentally, are not specifically attributed to this course within the framework of the degree program.

## Discussion and conclusion

UN Agenda 2030 seeks to ensure inclusive, quality education for all and promote lifelong learning (ODS 4), including explicitly higher education. Lifelong online learning—due to its compatibility with work, family, and personal situations—plays a key role in promoting employability education. In this sense, any study that seeks to analyze the effectiveness of the application of online educational techniques for the development of employability skills in online contexts is particularly relevant.

The study indicated that it is possible to promote authentic learning in an online environment. This result was consistent with previous research (Leppisaari et al., 2013; Rowe, 2016; Teras & Kartoglu, 2018) showing that the nine elements of Herrington and Oliver (2000) are also suitable for online contexts. The research also demonstrates that introducing authentic learning scenarios improve students’ perceptions about employability competences acquisition.

There is a wealth of literature on the application of the Herrington and Oliver model (2000) in face-to-face environments. However, the literature on this model in online environments is much more limited, both in relation to the diversity of courses in which it is applied, and the number of students involved in the related experiences. For this reason, our study focused on online programs, in asynchronous mode, and on a discipline that has not yet been addressed, business and management. A discipline that is relevant in the labor market due to the high number of jobs it represents. In addition, we involved more students in the experience than previous studies included in the literature. Moreover, it is important to note that a quantitative approach was used to assess the results obtained from the experience. An approach that had not been used previously in online contexts. This quantitative design was applied from the students' perspective. According to Gulikers et al., 2008, the students' perspective is key in the assessment of authenticity. The analysis showed that the students who participated in the study positively valued the redesign of the learning strategy, outlining a high degree of cognitive authenticity of the course.

At this point we want to reflect on the use of technologies in the recreation of authentic environments. The emergence of communication technologies that allow students to connect and collaborate with others around the world, or visualization technologies that lead to deep understanding and stimulate co-creation processes, or simulation tools that promote informed decision-making in complex situations, are clearly able to provide students with opportunities to experience and solve real-world problems. However, while technologies play an important role in implementing authentic learning in online contexts, they are not sufficient to ensure the quality of authenticity. Technological tools are a means of providing authenticity, not an end in themselves. The effectiveness of authentic learning scenarios depends on how the learning process is designed.

In this sense this research explained the redesign of a course in the field of finance, to give it greater cognitive authenticity. The redesign took advantage of the facilities provided by technology (especially through videos), but within a holistic and integrated vision of the student learning process. This experience can serve as an example and help for teachers in this discipline that may be prepared in terms of knowledge, attitude, and interest in using an authentic learning approach, but may lack the skills to fully implement it, as Baskaran and Abdullah (2020) identified.

The result of this experience has been a set of learning outcomes that can be useful to other teachers of the course, beyond the description of the concrete experience that can be recreated (as shown in Tables 4 and 5). We would like to underline that trying to fulfill all the attributes of ALS in the framework of a single course can sometimes be especially complex for the teacher, apart from involving a higher student dedication than the credit-load of the course. Therefore, the faculty must properly assess the level of depth at which each attribute of those indicated by Herrington and Oliver (2000) must be present in each of the courses in which an ALS is to be applied (for example, in relation to the level of collaborative work).

From the point of view of the teachers involved in the redesign, creating ALS is easier in advanced courses and closer to the insertion to the labor market of students. Advanced course students have a solid conceptual background that allows them to develop their learning more autonomously, and, additionally, the design of the course can more easily recreate a real work environment, linked to the learning results of the course, and at the same time, endowed with cognitive authenticity.

Research shows that, from the online student's perspective, ALS can contribute significantly to improving student competency profiles. This improvement can serve to boost employability, insofar as it strengthens specific competences in a particular field, as well as generic competences that, according to Ornellas et al. (2019), are the key to employability. It is also worth mentioning that, from the students' perspective, although the course remained results-oriented, it enabled them, above all, to improve their skills in terms of critical thinking, decision-making, problem solving and self-management.

So, we see that in a specific virtual learning environment, where personal circumstances and student schedules may not be compatible with internship, the introduction of authentic learning activities into the curriculum itself can provide a viable solution to strengthen student employability. And it can also provide an opportunity to rethink online learning methodology, making it more meaningful in cognitive terms, as well as being more in tune with labor market conditions.

To conclude, it should be noted that the study conducted had several limitations resulting from its design. First, because the results of the quantitative analysis were based on students' perceptions of employability. Students' responses were conditioned by their own personal characteristics (self-regulation level and self-efficiency, among others) and response patterns. Thus, it would be relevant to analyze the influence of the personal characteristics of the students on the answers provided. On the other hand, it would be interesting to complement the student's point of view with the perceptions of other stakeholders in the educational process, such as teachers and, especially, employers. Secondly, we must emphasize that the research was performed in a specific online context. Therefore, additional research is required to analyze to what extent these results can be extrapolated to other courses, disciplines, LMS and cultural contexts.

## Data Availability

It has not been possible to deposit the research data in a public repository.
